# Effects of the Truck Suspension System on Animal Welfare, Carcass and Meat Quality Traits in Pigs

**DOI:** 10.3390/ani7010005

**Published:** 2017-01-18

**Authors:** Filipe Antônio Dalla Costa, Letícia S. Lopes, Osmar Antônio Dalla Costa

**Affiliations:** 1 Programa de Pós-Graduação em Zootecnia, Faculdade de Ciências Agrárias e Veterinárias, University of São Paulo State UNESP-FCAV, Jaboticabal 14884-900, Brazil; filipedallacosta@gmail.com; 2 Grupo de Estudos e Pesquisas em Etologia e Ecologia Animal-ETCO, UNESP/FCAV, Jaboticabal 14884-900, Brazil; 3 Embrapa Swine and Poultry, BR 153, Km 110, Concórdia 89700-991, Brazil; leticia.lopes@embrapa.br

**Keywords:** blood metabolites, transport, truck suspension, stress, lesions, meat quality

## Abstract

**Simple Summary:**

Transportation is a complex stressor in which animals are exposed to a series negatively stimuli, such as vibration, new environmental conditions, variation in temperature and humidity, social mixing, noises among other poor factors, which can result in welfare problems and economic losses such as increased skin lesions, poorer pork quality traits. Transport stress may be reduced through a vehicle suspension system that provides a much smoother ride during transport, and consequently is less aversive to pigs. However, air suspension systems are more expensive and have bigger maintenance costs. This increase in transportation cost must be supported by the benefits from improvements in quality of freight transport; otherwise, the truckers will be paying unnecessarily for a similar or equivalent ride quality. Thus, finishing pigs were assessed after transport to slaughter by the same two double-decked trucks using two types of commercial vehicle suspension, leaf-spring and air suspension, to compare effects on blood cortisol and lactate at exsanguination, behaviour during lairage, and carcass (skin lesions) and pork quality traits. The use of leaf-spring suspension system negatively affects the welfare of pigs due to the increased carcass damage and resulted in poorer pork quality traits.

**Abstract:**

The objective of this study was to assess the effects of two types of commercial suspension (leaf-spring (LS) vs. air suspension (AS)) installed on two similar double-decked trucks on blood cortisol and lactate concentration, lairage behavior, carcass skin lesions and pork quality traits of 120 crossbred pigs. The suspension type neither influenced pig behaviour in lairage nor blood cortisol and lactate concentrations (*p* > 0.10). However, when compared with the AS suspension system, the use of LS increased the number of skin lesions in the back and thigh (*p* = 0.03 and *p* = 0.01, respectively) and produced thigh with lower pH_u_ (*p* < 0.001) and yellower colour (higher b* value; *p* = 0.03), and paler back muscles (subjective colour; *p* < 0.05), with a tendency to lower pH (*p* = 0.06). Therefore, the use air suspension system can improve carcass and meat quality traits of pigs transported to slaughter.

## 1. Introduction

The transport of pigs is an essential and inevitable process in the modern multi-site pig industry [1]. However, transportation is a stressful experience for pigs [2,3] that may result in economic losses due to mortality, carcass lesion and poor pork quality [2,4,5,6]. Within the effects of transport factors, the vibration of the truck plays an important role contributing to reduce the welfare of pigs during transport [7,8,9,10,11], travel sickness and stress [9,12], and increased skin lesions scores due to pigs’ slips and falls while the vehicle is moving [7,11,12].

Nowadays, in the transport sector, where every cent has to be taken into account, higher purchase and maintenance costs must be supported by the benefits from improvements in animal welfare and pork; otherwise the truckers will be paying unnecessarily for a similar or equivalent ride quality. The vibrations of the truck caused by the truck design and condition, poor suspension system, floor, driving style and road conditions are some of the factors affecting the welfare of pigs during transport [7,8,9,10]. However, truck vibration may be reduced through the suspension system, leaf-spring or air type [9,10,13,14], whose objective is to enhance the contact of the vehicle with the road surface and indirectly reduce vehicle vibrations [8].

Excessive exposure to vibrations have been shown to result in travel sickness and stress [9,12], and increased skin damage scores due to pigs’ slips and falls while the vehicle is moving [7,12]. However, when compared with the leaf-spring suspension, air suspensions proved to reduce vibrations of the truck up to five times more [13,15] and may thus provide livestock with more comfortable and smoother transport conditions [10,13]. According to Gebresenbet [13], a poor suspension system is one of the main factors causing vibration and loss of balance during livestock transport. Researches have mainly focused on effects of vibration on postural stability, behaviour during transport [9,12,13,14]; driving performance, truck speed, weight of shipment and road conditions on vibration level [9,13,15,16], and determination of vibration levels and frequencies of typical trucks during animal transport [10]. 

During transport, the level of truck vibration induces stress reactions such as increased heart rate, ectopic beats, cortisol and adrenocorticotropic hormone concentrations and postural behaviour changes that only occur under unusual environmental exposure [9,17,18]. The pork industry aims to obtain an optimal pork quality. However, the response to stressful situations increases the rate of glycolysis [19,20], which produces lactic acid early post mortem by an anaerobic metabolism [21]. Stressed pigs prior to slaughter show a rapid glycolysis of muscle glycogen with increased lactic acid production before the slaughter. Consequently, a lower pH is reached earlier while the carcass temperature still high and the pork quality traits are impaired due to the increase in protein denaturation [22,23,24]. Conversely, longer-term stress during hours until the slaughter moment reduces muscle glycogen stores and leads to a lower production of lactic acid post mortem. Hence, even after 24 h, the ultimate pH is still high, which reduces the pork quality traits [24,25]. Based on this, the physical activity due to difficulties in keeping balance and slips and falls during transport may affect the rate of glycolysis, resulting in an impaired meat quality. However, little is known about the effects of the suspension system on physiologic parameters of stress, behaviour and meat quality of pigs transported to abattoir. The aim of this study was to evaluate the effects of two types of commercial suspension systems, leaf-spring and air suspension, on the blood indicators of pigs’ welfare, behaviour during lairage, number of skin lesions in the back and thigh, and meat quality traits under commercial conditions.

## 2. Materials and Methods

All experimental procedures performed in this study were approved by the institutional animal care committee on the basis of the current guidelines of the São Paulo State University’s Animal Research Ethics Board (protocol number 6119-08).

### 2.1. Description of Study Site

The study was conducted in a commercial abattoir located in Southern Brazil (Rio Grande do Sul state) under federal inspection service. It is located 27°21′06″W (latitude) and 53°23′46″S (longitude) and at 531 m above sea level. The climate of the area is mild with an average rainfall of 20 mm for the studied period. However, there was no rain during the period from loading, transport, unloading until the slaughter. Transport trials were run during the winter of 2008 (June) at a relatively mild air temperature (to avoid thermal stress) with an average ambient temperature during the transport of 14 °C (range from 13 to 19 °C) and a thermo-humidity index of 57.3 (range from 55.7 to 66.1).

All farms were located within a radius of 56 km (54.4 ± 1.9 km) from the abattoir and consisted of only one growing-finishing facility with similar design, and capacity to finish over 450 pigs (±25) per cycle (170 days). Pigs were kept in pens (10.4 ± 0.4 pigs/pen) on concrete floor at an average density of 1.15 m^2^/pig. The topography of the area of transport from farms to abattoir was generally flat with a few steep slopes.

### 2.2. Animals, Loading Facilities and Handling of Pigs on the Farm

The number of skin lesions was visually counted on the left side of the body of each pig (120 pigs) used along all measurements, written down through a paper and pencil spreadsheet, and the proportion of pigs showing lesions at the farm on the day before transport, at unloading and slaughter was calculated. A total of 120 from 960 crossbred pigs (BW of 113 ± 1.1 kg) originating from five commercial swine growing-finishing farms were fasted for 12 h before loading and loaded in groups of 5–6 pigs by a trained loading crew using paddles and rattles always from 23 h to 24 h. At each farm, pigs had to climb an adjustable loading ramp (11.6 ± 0.5 m long), which could be set up to reach the bottom and the fixed upper deck (15° and 21° slope, respectively) of the truck. The loading order of the two trucks was switched in each farm (i.e., starting by the leaf-spring suspension truck in the first farm and by the air suspension truck in the next one consecutively). Two pigs per compartment for each treatment were used in this study (2 pigs/6 compartments/2 trucks/5 farms = 120 pigs). At each farm, the loading procedure took on average 20 min (±2 min).

### 2.3. Transport Conditions and Vehicle Features

Pigs were transported to a commercial slaughter plant using two similar double-decked trucks (Triel-HT, Erechim, Brazil) on each continuous journey (5 journeys) of 114 min (±22.7) on average and at a mean speed of 30 km/h on the same route. In each truck, pigs were distributed into 6 compartments (3 per deck) holding 16 pigs each at a density of 230 kg/m^2^. The average transport distance was 54.4 km (±1.9), consisting of 12.8 km (±3.9) on earth road and 41.6 km (±3.2) on paved road. The driver for each truck tested was the same throughout of the study and switched treatments each journey to reduce driver variations on the response of the pigs to transport as previously suggested in other studies [3,9,26,27].

The trucks had neither drinking nipples nor showers. Both trucks (Volkswagen 24.250 2008, Volkswagen Caminhões e Ônibus, Resende, Brazil) were fixed upper metallic deck trailer models equipped with six compartments (2.92 m length × 2.4 m width × 1 m height), loading surface of 42 m^2^ (6 compartments × 2.92 m × 2.4 m), drum braking system, natural ventilation, metallic open sides, and a grid cross slating floor (aluminum). Of the two trucks, one was equipped with a leaf-spring suspension system (LS; Volkswagen Caminhões e Ônibus, Resende, Brazil) and the other with an air suspension system (AS; Scania Latin America Ltda., São Bernardo do Campo, Brazil). The LS uses a semi-elliptic leaf spring system attached to the vehicle axle and consisting of several layers of flexible steel strips that are joined to cushion from impacts, whereas, the AS consists of valves, air pipes and woven and rubber-like material air-spring bags. The air originates from the truck air compressor and reservoir and pressurizes the air-spring bags that, similar to a spring, cushion from impacts during travel while increasing the contact of tires with the road surface.

### 2.4. Handling of Pigs at the Slaughter Plant

Pigs were immediately unloaded on arrival at the slaughter plant through an adjustable in slope metal ramp (5 m length, slope ≤ 15°) with anti-skid floor using paddles. Pigs were kept in separate pens (no social mixing; 5 m length × 4 m width × 1.5 m wall height) according to the treatment (LS vs. AS) for 3 h at a density of 0.6 m^2^/100 kg. All pens had a concreted floor and walls with solid metallic gate to conduct the pigs in/out a proportion of one nipple drinking to fifteen pigs with a flow of 2 L/min was respect. The ambient temperature at the abattoir ranged from 13–18 °C during the period of data collection, which was controlled with a sprinkling and forced ventilation system. At the end of lairage, pigs were driven to slaughter using paddles, boards and rattles and electrically stunned (head-only electrical stunning; 700 V, 1.3 A, 5 s; Valhalla, Stork RMS b.v., Lichtenvoorde, Holland) before exsanguination in the horizontal position within 30 s. This abattoir slaughters up to 2000 pigs/day and operated at 280 pigs/h during the data collection of this study.

### 2.5. Behavioral Observations during Lairage

The behavior of pigs in lairage was assessed in each pen by one trained observer using the scan sampling method consisting of direct observations at 15 min intervals along the 3 h period and analyzed by the hour. The recorded behaviors are listed in Table 1. The proportion of pigs expressing each behaviour was calculated per period of 60 min.

### 2.6. Blood Parameters

A sub-sample of 120 pigs (2/compartment/truck or treatment, totalizing 12 pigs/truck or treatment/replicate or journey) was used for the analysis of blood stress indicators in the bleeding blood. Blood samples (10 mL) were collected in a tube (Vacuplast, Cral Artigos para Laboratório Ltd., São Paulo, Brazil) for cortisol analysis. Another 2 mL of blood was collected in a tube containing 3.0 mg of sodium fluoride and 6.0 mg of Na2EDTA solution to extract plasma for lactate concentration analysis. Samples were immediately centrifuged at 4 °C for 12 min at 1400 *g*. Plasma was transferred into 1.5 mL Eppendorf tubes and stored at −80 °C until lactate concentration analysis. Serum samples were kept at room temperature (~23 °C) for 1 h before refrigeration at 4 °C. The following day, serum samples were centrifuged at 4 °C for 12 min at 1400 *g*, the supernatant was transferred into 1.5 mL Eppendorf tubes and stored at −80 °C until analysis. Plasma lactate levels were measured using a commercially available kit (Lactat PAP Enzyme Farbtest, Rolf Greiner Biochemica, Flacht, Germany) and their plasma concentration determined with a microplate reader. The quantitative determination of cortisol was made using a commercial kit (Coat-A-Count Cortisol Kit, Diagnostic Products Corporation, Los Angeles, CA, USA) with a microplate reader and expressed in ng/mL. The intra-assay CV was 26.08% and 22.43% for lactate and cortisol concentration, respectively.

### 2.7. Carcass Handling and Skin Lesion Assessment

After slaughter, carcasses were eviscerated, split and chilled (1–4 °C for 24 h) according to standard commercial practices. In the cooler, lesions were assessed on each left carcass side and classified as fighting-type lesions (1 = less than 10 lesions; 2 = 11 to 20 lesions; and 3 = greater than 20 lesions) or mounting-type lesions (score 1 = less than 5 lesions; 2 = 6 to 10 lesions; and 3 = greater than 10 lesions) by visual assessment of shape and size according to the photographic standards of the Institut Technique du Porc (ITP) [28] as described by Faucitano [29]. According to the ITP scale, lesions due to biting during fighting are 5 to 10 cm in length, comma shaped, and concentrated in high number in the anterior (head and shoulders) and posterior (thigh) regions of the carcass. Long (10 to 15 cm), thin (0.5- to 1-cm-wide), comma shaped lesions densely concentrated on the back of pigs caused by the fore claws were classified as mounting type lesions. And large dark brown rectangular marks usually found on the middle, back and hind regions were classified as handling lesions.

### 2.8. Meat Quality

Meat quality was evaluated in the *Longissimus dorsi* (back) and *Semimembranosus* (thigh) muscle of the same pigs previously evaluated. Muscle pH was assessed at 45 min (pH_i_) and 24 h (pH_u_) post mortem using a pHmeter (HI 8314 model, Hanna Instruments, São Paulo, Brazil) fitted with a spear tip electrode (HI 1217D, Hanna Instruments, São Paulo, Brazil) and an automatic temperature compensation probe (Tec 530, Hanna Instruments, São Paulo, Brazil) by insertion into the *Longissimus dorsi* (LD; between the 13th and 14th rib) and *Semimembranosus* (SM) muscles. At 24 h of slaughter, objective and subjective colour, and drip loss measurements were taken in the LD and SM muscles. Instrumental colour (L*, a* and b* values) was measured using a Minolta Chromameter (CR-400; Minolta Camera Ltd., Osaka, Japan) equipped with a 25 mm aperture, 0° viewing angle, and D65 illuminant. Visual colour was evaluated through the Japanese Pork Color Standards (JPCS; ranging from 1 = pale to 6 = dark color) [30]. Percentage of drip loss was measured using a modified EZ-driploss method [31]. Following this method, at 24 h post mortem, two muscle cores samples of 25 mm diameter were taken from the center of 2.5 cm thick LD (at the level of the 13th/14th last rib) and SM muscle chops using a specific drip loss knife, weighed and placed into funnel-shape plastic drip loss containers (KABE Labortechnik, Umbrecht-Elsenroth, Germany) and stored for 48 h at 4 °C. Muscle core samples were carefully collected from their containers using a tweezers after the 48 h storage period, surface moisture of cores were carefully dabbed prior being reweighed, and then drip loss percentage was determined by dividing the difference between initial and final core weights by the initial core weight. The back and thigh muscles were classified according to pH_u_, drip loss and light reflectance (L*) variation (Table 2). To assess cooking losses, four LD muscle chops (150 g each) were individually vacuum-packed in heat resistant sealed plastic bags (nylon polyethylene bag 16 × 30 × 0.1 cm) 10 micron thick with a vacuum of 0.8 bar with a double seal using a DZ-4000 vacuum packer machine (Cetro Solutions in packing, Taiwan, China), and cooked in water bath at 80 °C for 1 h. After cooking, chops were then placed on absorbent paper to remove surface moisture and weighed when the internal temperature reached 20–25 °C. The weight loss was calculated by difference between initial and final weight [32]. The same LD muscle chops were then cooled at 2–4 °C for 12 h and used for the determination of Warner-Bratzler shear force. Five rectangular cores (1 × 1 × 2 cm), parallel to the longitudinal orientation of the muscle fibers, were taken in each chop and analyzed using a Warner-Bratzler device attached to a TAXT2i Texture Analyzer (Stable Micro Systems, Survey, UK).

### 2.9. Statistical Analysis

Data was analysed as randomized complete block design to check effects of treatments by analysis of variance in ANOVA. Values of blood cortisol and lactate concentration were log-transformed (Ln) for data normalization before analysis. Frequencies of lesions were transformed and expressed as the square root of (x + 1). The model included effects of block (two decks: upper and lower × three position of compartments: front, middle, rear), day of transport (1, 2, 3, 4 and 5), farm, treatments, interaction between farm and block, and error (correspondent to randomized variation on the observations in the day of farm and vehicle suspension system), supposedly homoscedastic, independent and normally distributed. Variance analysis using GLM SAS (2003) was applied to study the effects of truck suspension using the group as experimental unit for the analysis of behaviour data, and the individual as the experimental unit for the analysis physiological and meat quality data, and transport was the adopted repetition. For the analysis of pig behaviour data during the lairage period, the experiment was conducted according to a randomized block design (farm) with two treatments (trucks) in the plot and 3 repeated measures over times (1st, 2nd, and 3rd hour of lairage). The likelihood ratio and chi-square tests were used to compare the skin lesion-type categories. The tests were performed using the FREQ procedure of SAS (2003) with Student’s *t* test protected by the significance of the F test for mean comparison. A probability level of *p* < 0.05 was chosen as the limit for statistical significance in all tests and probability levels of *p* ≤ 0.10 were considered as a tendency. There was no significant effect and interaction of farm, block and farm, or block in any of the studied variables (*p* > 0.10).

## 3. Results and Discussion

### 3.1. Behaviour during Lairage

Overall, in this study lairage time had an effect on pig behaviors (*p* < 0.05), with the proportion of standing, walking and sitting pigs decreasing with time after the first hour of lairage and that of pigs lying down increasing to almost all pigs at the end of lairage. However, no effect of the truck suspension system, deck and compartment position was observed on these behaviours in this study (*p* > 0.10; Table 3).

After the first hour of lairage, there was a significant reduction in the expression of standing, walking, and sitting; and consequently an increase in the frequency of the pigs lying down. After one hour of lairage, more than 80% of pigs were lying, and practically all pigs were lying within 3 h. The level of vibration is affected by the vehicle suspension system [9], which may hamper pigs to keep their posture and prevent them lying down during transport. Swaying behaviour and loss of balance can also increase with transport time [9]. In this condition, due to the physical exhaustion and stress [13,26,34], pigs would prefer settling and lying down to rest instead of doing other activities (i.e., standing, walking and fighting) during the lairage, as found in other studies [35]. However, other transport conditions (i.e., road conditions, proper density, and relatively mild air temperature-driven behaviour) used in this study may have compensated the vibration level and reduced any effects on the pigs’ behaviour during lairage [9,10,26,35]. Indeed, both drivers had already applied for training of animal welfare and defensive driving, which may have reduced potential differences of truck vibration on pigs’ behaviour during lairage [9,26].

### 3.2. Physiological Response

In this study, the type of suspension system had no effects on blood cortisol (9.16 ± 0.49 vs. 7.95 ± 0.54 μg/dL for LS and AS, respectively; *p* > 0.10) and lactate levels (14.33 ± 0.71 and 14.26 ± 0.86 mmol·L^−1^ for LS and AS, respectively; *p* > 0.10) at exsanguination. Vibration values are greater in the upper deck in vertical and lateral position, while the lower deck presents greater vibration on driving direction [9]. Even with these differences, no effects of deck and compartment position were found (*p* > 0.10). Differently from a previous study, increased blood cortisol levels were observed in piglets subjected to vibrations [7]. After the beginning of vibration simulation, Perremans et al. [18] found a quick increase in blood cortisol levels, which returned to baseline levels within one hour after the end of stimulus. As blood lactate concentration returns to basal levels within 2 h after physical exercise [36,37], the lack of effect of truck suspension system on blood lactate may indicate that all pigs, regardless of the transport conditions, recovered from handling and transport stress thanks to the adequate lairage conditions (i.e., sufficient space allowance to lie down and ambience). Nonetheless, in both groups, the blood lactate levels at exsanguination were greater than the resting level of blood lactate for market-weight pigs (>4 mM; [38]), reflecting a general state of fatigue in all pigs transported. The CV values found are high, which may reflect an individual effect on these variables, and contributed to the lack of significant differences between treatments.

### 3.3. Skin and Carcass Lesion

The transport conditions of pigs can impact directly on carcass lesions. In agreement with previous studies [3,5,39], in this study, the overall proportion of pigs presenting skin lesions increased from farm to slaughter (29% to 62%) as a result of the additive effects of loading, transportation, unloading and lairage.

There was a significant effect of truck suspension system on the number of carcass lesions located in the back and thigh (*p* ≤ 0.05; Table 4). Pigs transported in LS truck showed a greater number of carcass lesions caused by animal mounting than in LS (*p* ≤ 0.05; Table 4). A tendency to more carcass lesions caused by handling was observed (*p* ≤ 0.10; Table 4). Aradom et al. [9] found different vibration values according to the deck position (upper vs. lower). However, in this study, different from Barton-Gade et al. [13] and Dalla Costa et al. [5], who found higher number of bruises in pigs transported in the rear transport compartment, no effect of the deck (upper vs. lower) or compartment position (front, middle, rear) on number of skin lesions in the back and thigh was found (*p* > 0.10 for both).

During transport, pigs may have difficulties in keeping posture and, consequently, lose balance and fall. Because pigs tend to stand up during the uncomfortable conditions of transport, especially during short journeys [40,41], increased carcass lesions are observed due the loss of balance and slips and falls [42]. Rough driving styles and poor suspension system are the main factors causing vibration and loss of balance during animal transport [16]. The truck equipped with AS may have provided smoother transport conditions for the pigs [8,9,10], and consequently, reduced the number of pigs slipping and falling, stepping on each other and striking themselves against the walls of the truck compartments [18]. Based on this, pigs facing better transport conditions can have a lower incidence of lesions as a result of exposure to a lower frequency of loss of balance. The cumulative effect of slips and falls during transport is likely to explain the increased number of lesions observed in the back and thigh. When a pig slips and falls down and has some difficulties in standing up again due to the vibration level and loss of balance, there is a considerable chance of other pen mates tramp this pig which may contribute to increasing the number and severity of skin lesions classified as mounting. Indeed, the fall of one pig can results in slips and falls of other pen mates from the same compartment. The stocking density adopted was within the regulation of EU for both trucks (230 kg/m^2^) [43]. However, the greater number of carcass lesions classified as mounting observed in LS truck is suggestive of more pigs stepping on each other in order to keep balance during the transport than in the AS truck. Indeed, the fall of one pig can cause the loss of balance or fall of other pen mates’ pigs. Overall, a loss of balance and fall means welfare problems in the form of bruises, injuries and physical fatigue.

A confounding effect of handling during loading/unloading, mixing unfamiliar pigs, social conflicts and driving should be considered with regard to carcass lesions. However, in order to avoid this problem, the experimental design of the study was done to make both groups receive the same treatments during all steps of preslaughter handling, and so, they have the same random influences. All procedures of loading and unloading were done by a trained crew, which was previously advised to have the same behaviour in all loading and unloading in order to avoid any interference on pigs’ behaviour and welfare between treatments. Indeed, all procedures were watched by a technical team, aiming to identify any critical point during these phases and clear difference on the handling between treatments, but no differences were noticed. In order to reduce the response to transport driving behaviour, the loading order and driver used were switched in each treatment, and both drivers used here had previously took training of animal welfare and defensive driving [9,26,27]. Thus, based on this, even with the effect of mixing that was not possible to be avoided under commercial conditions and influence both groups equally, the differences found were mainly due to the effects of treatments.

### 3.4. Meat Quality

There was no significant effect of suspension, deck and compartment position system on meat quality classification (*p* > 0.10; Table 5). However, pigs transported by LS truck had lower JPCS scores in *Longissimus dorsi*, and lower pH_u_ mean and higher value of color b* in *Semimembranosus* muscles than pigs transported by AS truck (Table 6). The pH_u_ mean in *Longissimus dorsi* and JPCS score in *Semimembranosus* tended to be lower in pigs transported by LS truck. In agreement with this study, Warriss et al. [44] reported potential effects of vibration on muscle pH, and glycogen reserves in broiler chickens. When stimulated for 3 h of vibration, the pH in both white (*Pectoralis superficialis*) and red (*Biceps femoris*) muscles decreased.

The variation in pork pH_u_ and JPCS score may be explained by the greater occurrence of mounting behaviour found in LS truck, as observed in carcass lesions (Table 4). Pigs transported in LS truck had a pH_u_ value lower than 5.5 in *Loguissimus dorsi*, which may indicate a mild pork quality defect. Conversely, even in red muscles such as the *Semimembranosus*, values of pH_u_ did not exceed the threshold of 6.3, which when exceeded is indicative of dark, firm and dry (DFD) pork [13]. Except for the visual colour score, the Minolta L* value, which is an indicator of colour lightness, was not affected by truck suspension system in this study. This lack of difference in Minolta L* value may be caused by the confounding effect of the light reflectance of marbling fat, which does not influence visual colour score. Because of fat colour reflectance, higher marbling scores may have resulted in higher L* values, while visual colour sores are not influenced by marbling scores [44,45]. The distribution shown in Table 5 is similar to other studies [3,46] that evaluated the meat quality in pig abattoirs in Brazil under very similar conditions of this study. The most desirable meat quality classification for the pork chain is red, firm and non-exudative (RFN), which had the highest incidence in agreement to the literature [3,46]. The variation in meat quality classification depends on the level of pre-slaughter stress, which can affect the muscle glycogen level. Stressful conditions at the moment of slaughter increase the muscle metabolism continually after death, and hence, a lower pH_i_ (pH_i_ < 6.0) is found in a drop rate increased by two to four times due to the increased production of protons and lactate in the early post mortem period [47,48]. Conversely, stress during different stages of pre-slaughter period may lead to low glycogen content at the time of slaughter, which reduces the amount of protons and lactic acid produced, and then, restricts the pH fall [49]. According to Henckel et al. [48], the pH_u_ values only increase due to muscle glycogen content lower than 53 μmol/g at slaughter. Usually, these phenomenon results in increased PSE and DFD meat, respectively. However, the incidence of PSE defect in pork was low, and the no incidence of DFD defect was found in this study.

## 4. Conclusions

The current study indicates that the truck suspension system can affect the welfare of pigs in the form of injuries, which results in reduced pork quality traits and losses to the pork chain. Thus, based on these results, the welfare and meat quality traits of pigs were better in AS truck than LS. The authors hope these results will stimulate further researches and lead to development, identification and use of technologies to improve welfare of animals during transport and, consequently, make the pig industry more sustainable.

## Figures and Tables

**Table 1:** Ethogram of pigs’ behavior during lairage.

Behaviour	Description
Standing	Pig on all fours limbs extended and stopped.
Walking	Pig on all four limbs and moving around.
Seated	Pig seated on the caudal region of body with forelimbs extended.
Lying down	Pig with lateral or ventral body surface in contact with the floor and without support of the fore and hindquarters.
Fighting	Two or more pigs performing a sequence of aggressive physical contacts for more than 3 s, such as biting, head knocking, pushing and shoving each other, with no grater intervals than 10 s.

**Table 2:** Meat quality classification [3,33].

Classes ^1^	pH_u_	Driploss	L*
PSE	<6.0	>6.0	>50
RSE	<6.0	>6.0	45–50
PFN	<6.0	<6.0	>50
RFN	5.5–6.1	<6.0	<50
DFD	≥6.1	<3.0	<44

**^1^** PSE: pale, soft, exudative; RSE: red, soft, exudative; PFN: pale, firm, non-exudative; RFN: red, firm, non-exudative; DFD: dark, firm, dry.

**Table 3:** Effects of truck suspension system on behavior **^1^** of pigs during lairage.

Behaviour (%)	Period (h)	Truck Suspension System ^2^		Mean
		AS	LS	
**Standing**	1	47.08 ± 10.32 **^A^**	49.58 ± 6.23 **^A^**	48.33 ± 5.70 **^A^**
	2	12.08 ± 4.08 **^B^**	12.08 ± 7.05 **^B^**	12.08 ± 3.84 **^B^**
	3	5.83 ± 3.39 **^B^**	1.25 ± 0.83 **^B^**	3.54 ± 1.81 **^B^**
	Mean	21.67 ± 6.03	20.97 ± 6.25	
**Lying down**	1	40.42 ± 14.53 **^A^**	41.67 ± 8.36 **^A^**	41.04 ± 7.91 **^A^**
	2	83.75 ± 4.99 **^B^**	82.50 ± 10.43 **^B^**	83.13 ± 5.45 **^B^**
	3	91.67 ± 4.80 **^B^**	97.92 ± 1.61 **^B^**	94.79 ± 2.60 **^B^**
	Mean	71.94 ± 7.81	74.03 ± 7.58	
**Seated**	1	5.42 ± 1.56 **^A^**	5.83 ± 1.67 **^A^**	5.63 ± 1.08 **^A^**
	2	2.92 ± 1.06 **^B^**	2.92 ± 1.06 **^B^**	2.92 ± 0.71 **^B^**
	3	1.67 ± 0.78 **^B^**	0.83 ± 0.83 **^B^**	1.25 ± 0.56 **^B^**
	Mean	3.33 ± 0.7	3.19 ± 0.86	
**Walking**	1	5.42 ± 2.60	2.92 ± 1.56	4.17 ± 1.49 **^A^**
	2	1.25 ± 1.25	0.83 ± 0.83	1.04 ± 0.71 **^B^**
	3	0.00 ± 0.00	0.00 ± 0.00	0.00 ± 0.00 **^B^**
	Mean	2.22 ± 1.08	1.25 ± 0.64	
**Fighting**	1	1.67 ± 1.67	0.00 ± 0.00	0.83 ± 0.83
	2	0.00 ± 0.00	1.67 ± 1.67	0.83 ± 0.83
	3	0.83 ± 0.83	0.00 ± 0.00	0.42 ± 0.42
	Mean	0.83 ± 0.60	0.56 ± 0.56	

**^1^** See Material and methods (Table 1) for more details; **^2^** LS Truck = Leaf-spring suspension system; AS Truck = Air suspension system; **^A^**^,^^**B**^ Different letters indicate statistically significant differences (*p* ≤ 0.05) by Student’s *t* test protected by the significance of the F test in the column.

**Table 4:** Number **^1^** of carcass lesions (mean ± SE) in pigs transported by trucks with different suspension systems.

Site of Carcass	Truck Suspension System ^2^		*p*-Value
	LS Truck	AS Truck	
Back	5.32 ± 0.62	3.73 ± 0.41	0.03 *
Thigh	2.26 ± 0.28	1.47 ± 0.15	0.01 *
Shoulder	3.22 ± 0.66	3.35 ± 0.88	0.90
**Lesion Type**			
Handling	2.78 ± 0.28	2.18 ± 0.23	0.08 **^†^**
Fight	5.80 ± 1.13	4.35 ± 1.09	0.32
Mounting	2.06 ± 0.22	1.38 ± 0.18	0.02 *

**^1^** Number of lesions expressed in root of (x + 1); ^**2**^ LS Truck = Leaf-spring suspension system; AS Truck = Air suspension system; **^†^** *p* < 0.10, * *p* < 0.05 indicate significant statistical difference.

**Table 5:** Meat quality classification **^1^** according to effect of truck suspension system (LS or leaf-spring suspension vs. AS or air suspension system) as assessed in the *Longissimus dorsi* and *Semimembranosus* muscles **^2^**.

Muscle	Classes ^3^	Truck Suspension System	
		LS	AS
*Longissimus dorsi* (Back)	PSE	6 (10.00%)	5 (8.33%)
	RSE	20 (33.33%)	17 (28.33)
	PFN	4 (6.67%)	4 (6.67)
	RFN	30 (50.00%)	34 (56.67)
	DFD	-	-
*Semimembranosus* (Thigh)	PSE	2 (3.33%)	2 (3.33%)
	RSE	22 (36.67%)	16 (26.67%)
	PFN	3 (5.00%)	5 (8.33%)
	RFN	33 (55.00%)	37 (61.67%)
	DFD	-	-

**^1^** See Material and methods (Table 2) for more details; **^2^** Number and percentage of back and thigh muscles classified in each treatment; **^3^** PSE: pale, soft and exudative; RSE: red, soft and exudative; PFN: Pale, Firm, non-exudative; RFN: red, firm and non-exudative; DFD: dark, firm and dry.

**Table 6:** Values (mean ± SE) of pork quality parameters of pigs transported by trucks with different suspension system.

Muscle	Variable ^1^	Suspension System ^2^		*p*-Value
		LS Truck	AS Truck	
*Longissimus dorsi* (Back)	pH_1_	6.41 ± 0.03	6.36 ± 0.03	0.1910
	pH_u_	5.45 ± 0.07	5.56 ± 0.04	0.0641 **^†^**
	T_1_	28.50 ± 0.19	28.62 ± 0.14	0.5138
	L*	47.57 ± 0.31	47.30 ± 0.29	0.4959
	a*	7.35 ± 0.17	7.47 ± 0.18	0.5129
	b*	−0.43 ± 0.20	−1.96 ± 0.96	0.1040
	JPCS	2.68 ± 0.07	2.97 ± 0.09	0.0065 *
	DL, %	4.86 ± 0.31	5.13 ± 0.27	0.5424
	WLC, %	39.50 ± 0.19	39.99 ± 0.37	0.1773
	SS, Kgf	4.32 ± 0.19	4.53 ± 0.13	0.4015
*Semimembranosus* (Thigh)	pH_1_	6.52 ± 0.02	6.52 ± 0.03	1.0000
	pH_u_	5.53 ± 0.03	5.61 ± 0.04	0.0003 *
	T_1_	28.76 ± 0.19	28.82 ± 0.13	0.7698
	L*	46.75 ± 0.26	46.43 ± 0.34	0.3587
	a*	6.83 ± 0.23	6.96 ± 0.17	0.5957
	b*	−1.29 ± 0.24	−1.73 ± 0.17	0.0318 *
	JPCS	3.01 ± 0.07	3.17 ± 0.08	0.0916 **^†^**
	DL, %	2.55 ± 0.23	2.70 ± 0.23	0.6489

**^1^** pH_1_ = pH 45 min post mortem; pH_u_ = pH 24 h post mortem (pH_u_); T_1_ = muscle temperature at 45 min post mortem; L = luminosity; a = red color; b = yellow color; JPCS = Japanese Pork Color Standards; DL = Drip loss; WLC = water loss by cooking; SS = shearing strength; **^2^** LS Truck = Leaf-spring suspension system; AS Truck = Air suspension system; **^†^** *p* < 0.10, * *p* < 0.05 indicate significant statistical difference in the same line by Student’s *t* test, protected by the significance of the F test.
